# Supplementary material for: A Novel Aging-Related Prognostic lncRNA Signature Correlated with Immune Cell Infiltration and Response to Immunotherapy in Breast Cancer
Source: Molecules. 2023 Apr 7;28(8):3283. doi: 10.3390/molecules28083283 (PMC10141963; doi:10.3390/molecules28083283)
Supplement: Supplementary file 1 [file molecules-28-03283-s001.zip › Table S2.pdf]

Supplementary Table S2

Univariate Cox regression analyses of the prognostic genes in BC patients.

id	HR	HR.95L	HR.95H	pvalue
MCF2L-AS1	0.767610608	0.608996782	0.967535565	0.025128796
SH3BP5-AS1	0.583116232	0.406558082	0.836349234	0.003377231
MIR205HG	0.854099401	0.75853268	0.961706471	0.00919021
LINC00667	0.692148585	0.517206543	0.926263734	0.013315183
USP30-AS1	0.661116648	0.500231974	0.873745072	0.003630733
PCED1B-AS1	0.749424923	0.587263147	0.956364651	0.020417087
OTUD6B-AS1	1.651814535	1.229200756	2.219727936	0.000872621
NDUFV2-AS1	0.547234793	0.349565683	0.856679972	0.00837804
LINC00852	0.697563386	0.496687084	0.979680554	0.037669616
MAPT-AS1	0.700658691	0.570506949	0.860502404	0.000691559
PRR34-AS1	0.673652928	0.515970006	0.879524511	0.003690224
DLGAP1-AS1	0.647452242	0.477642793	0.877631592	0.005094296
FGF14-AS2	0.769959853	0.592988185	0.999747029	0.049778471
EGOT	0.770570295	0.635028521	0.935042379	0.008280812
TP53TG1	0.805265389	0.666201034	0.97335836	0.025147315
ITGB2-AS1	0.752895963	0.578668329	0.979580708	0.034549868
NIFK-AS1	0.39397126	0.22160644	0.700400919	0.00150875
LINC00926	0.569817352	0.377494322	0.860123706	0.007424237
C6orf99	1.264130005	1.036053174	1.542415687	0.020951186
DLG5-AS1	0.622523445	0.452078254	0.857230881	0.003687709

Multivariate Cox regression analyses of the prognostic genes in BC patients.

id	HR	HR.95L	HR.95H	pvalue
MCF2L-AS1	0.517837172	0.220190825	1.217831567	0.131479545
USP30-AS1	0.401234907	0.164683399	0.977569395	0.044442415
OTUD6B-AS1	4.116907829	0.766385196	22.11541945	0.098989825
MAPT-AS1	0.419937612	0.203851908	0.865077003	0.018621329
PRR34-AS1	0.337598706	0.095085965	1.198629966	0.093013979
DLGAP1-AS1	0.221488665	0.065282256	0.751463434	0.015589737
