# Supplementary material for: A Novel Aging-Related Prognostic lncRNA Signature Correlated with Immune Cell Infiltration and Response to Immunotherapy in Breast Cancer
Source: Molecules. 2023 Apr 7;28(8):3283. doi: 10.3390/molecules28083283 (PMC10141963; doi:10.3390/molecules28083283)
Supplement: Supplementary file 1 [file molecules-28-03283-s001.zip › Table S3.pdf]

Supplementary Table S3. Clinical characteristics of BC patients in the two cohorts

Characteristic	TCGA patients (n = 1022)	GSE20685 patients (n=327)
**Age, n (%)**		
≤65	736 (72.0)	305 (93.3)
>65	286 (28.0)	22 (6.7)
**WHO-Stage, n (%)**		
I	179 (17.5)	37 (11.3)
II	577 (56.5)	34 (10.4)
III	226 (22.1)	41 (12.5)
IV	18 (1.8)	215 (65.8)
Unknow	22 (2.1)	0 (0)
**AJCC-T stage, n (%)**		
T_1	275 (26.9)	101 (30.9)
T_2	581 (56.9)	188 (57.5)
T_3	128 (12.5)	26 (7.9)
T_4	35 (3.4)	12 (3.7)
Unknow	3 (0.3)	0 (0)
**AJCC-N stage, n (%)**		
N_0	480 (47.0)	137 (41.9)
N_1	345 (33.7)	87 (26.6)
N_2	109 (10.7)	63 (19.3)
N_3	71 (6.9)	40 (12.2)
Unknow	17 (1.7)	0 (0)
**AJCC-M stage, n (%)**		
M_0	847 (82.9)	319 (97.6)
M_1	20 (1.9)	8 (2.4)
Unknow	155 (15.2)	0 (0)
