# Supplementary figures and images for: A Novel Aging-Related Prognostic lncRNA Signature Correlated with Immune Cell Infiltration and Response to Immunotherapy in Breast Cancer
Source: Molecules. 2023 Apr 7;28(8):3283. doi: 10.3390/molecules28083283 (PMC10141963; doi:10.3390/molecules28083283)

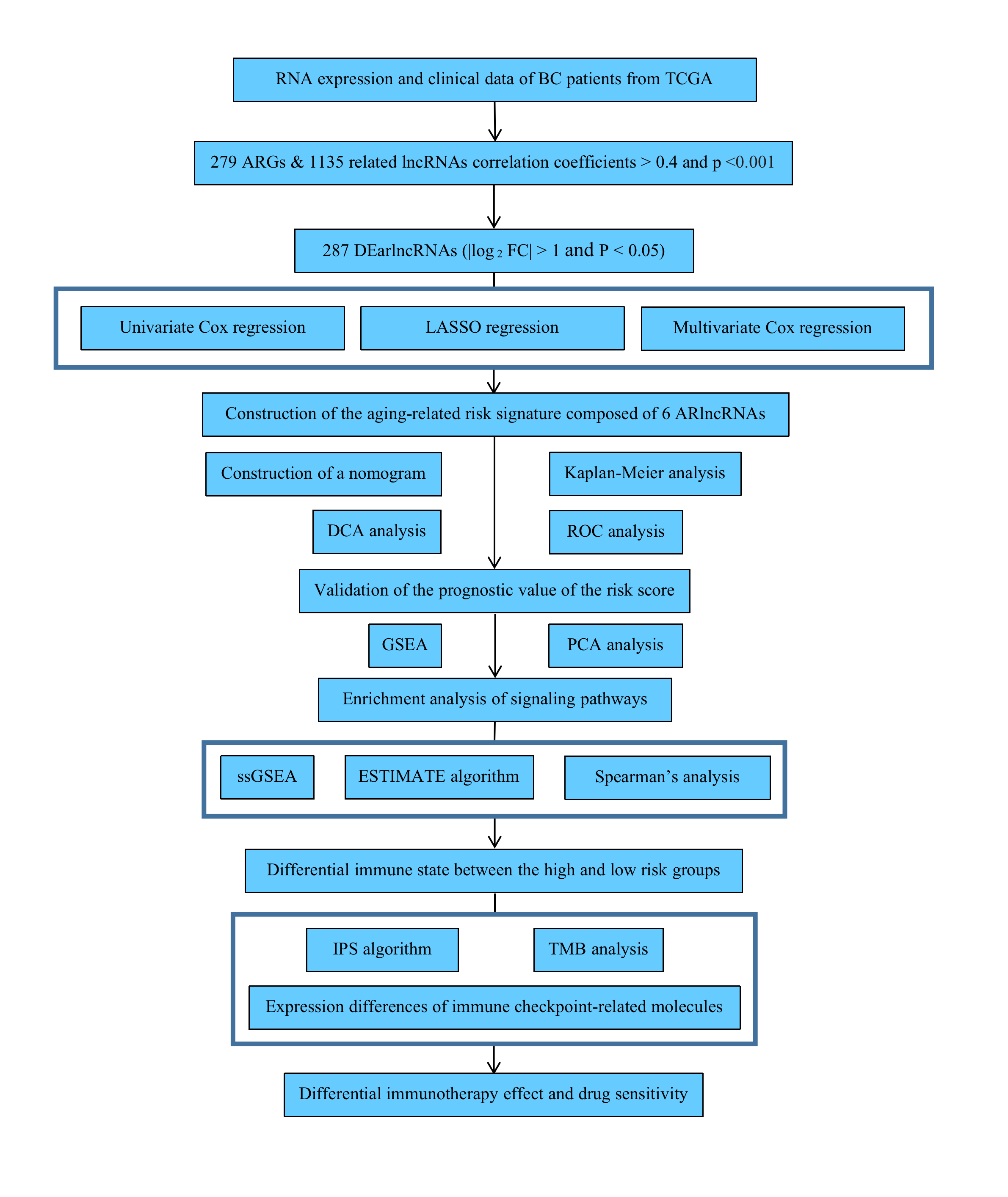

Supplement: Supplementary file 1 [file molecules-28-03283-s001.zip › Figure S1.tif]

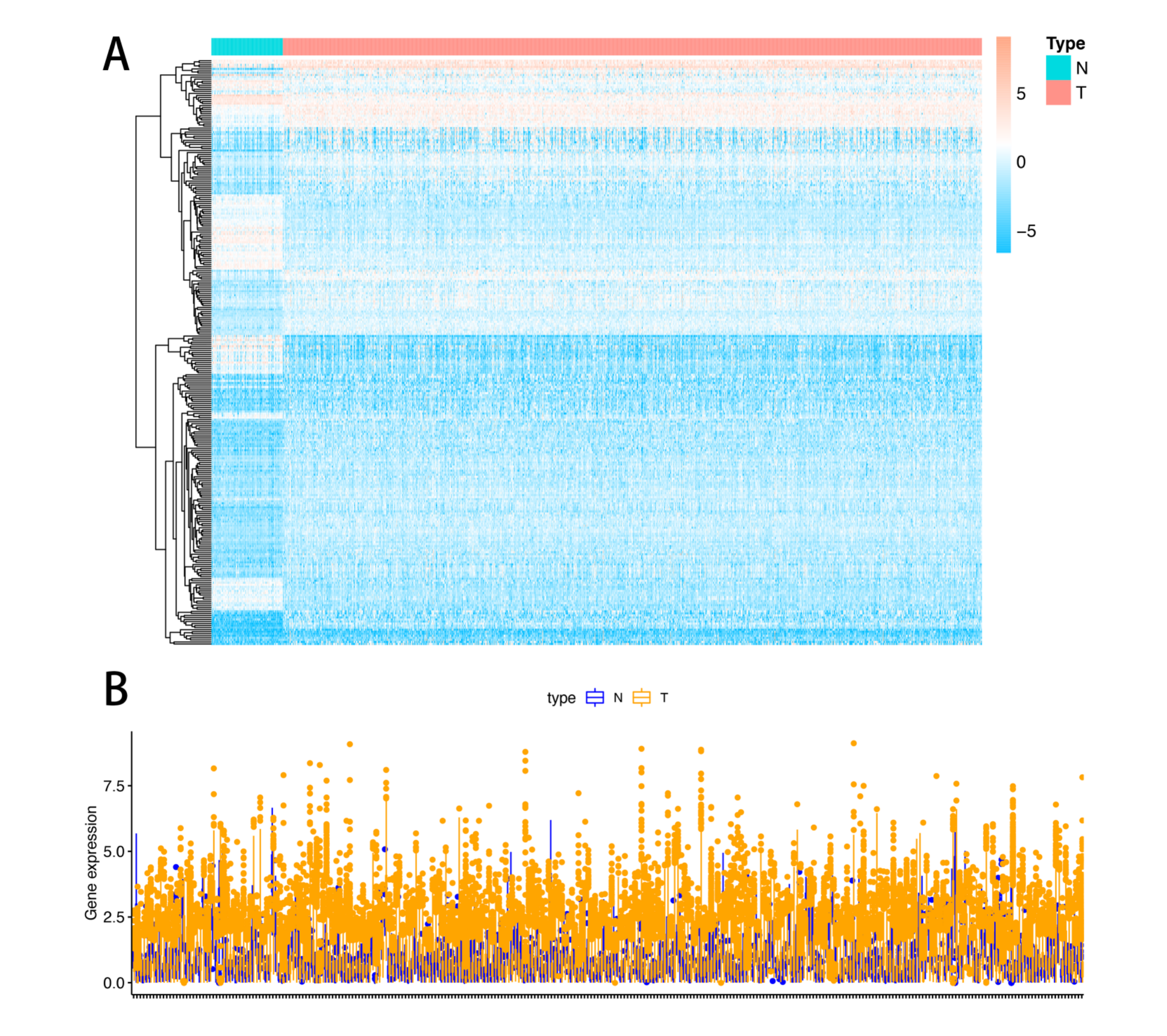

Supplement: Supplementary file 1 [file molecules-28-03283-s001.zip › Figure S2.tif]

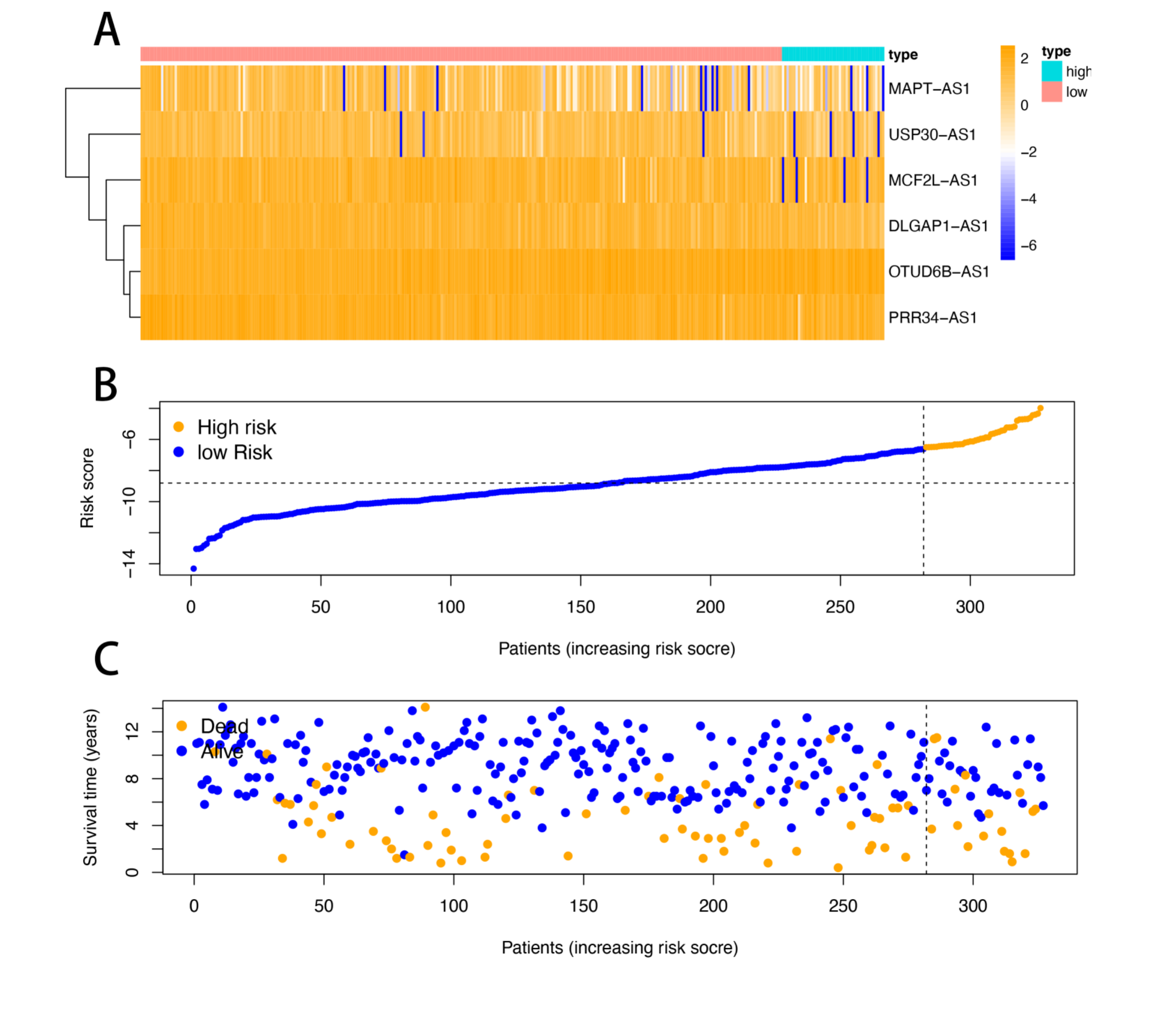

Supplement: Supplementary file 1 [file molecules-28-03283-s001.zip › Figure S3.tif]

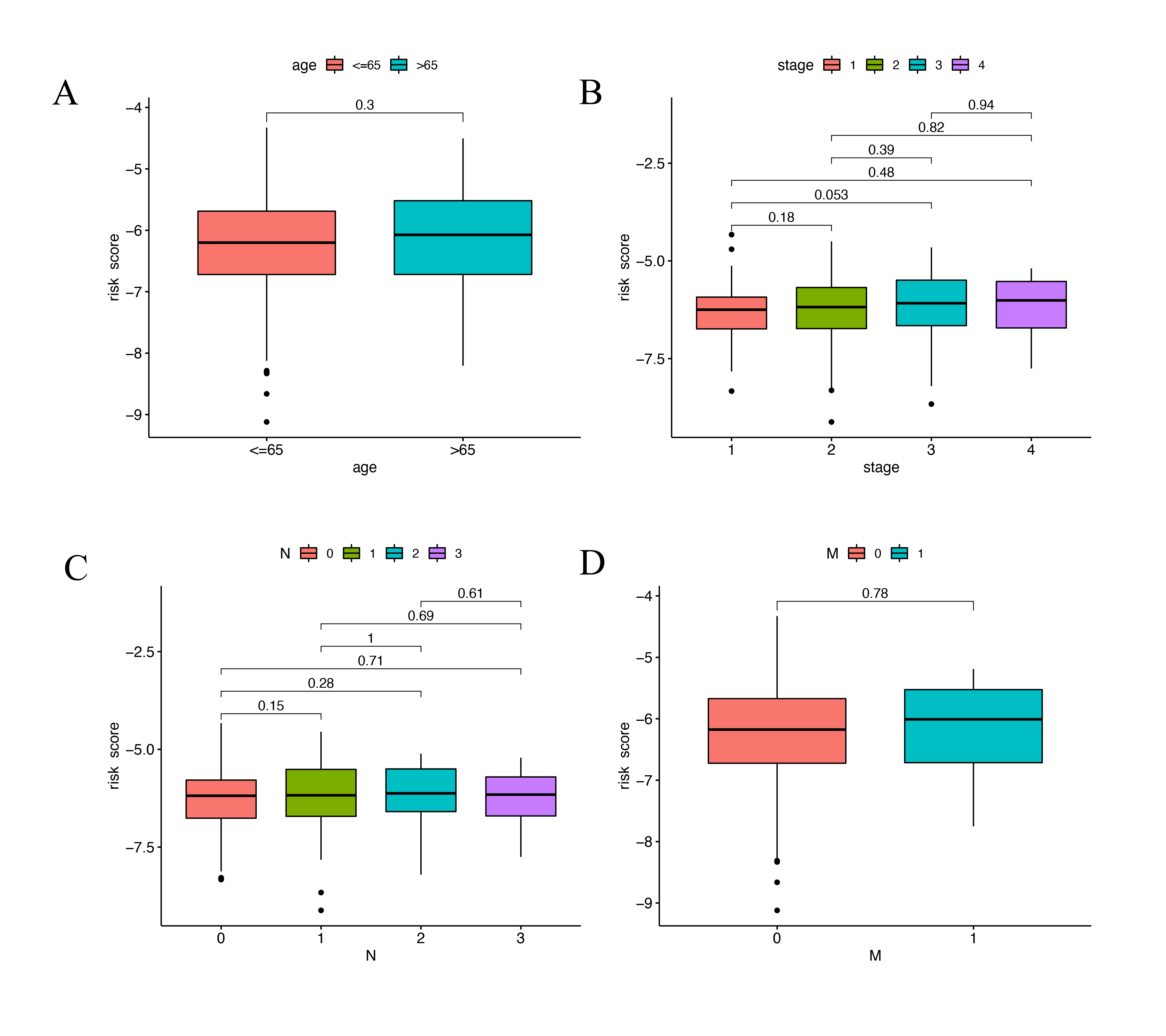

Supplement: Supplementary file 1 [file molecules-28-03283-s001.zip › Figure S4.tif]

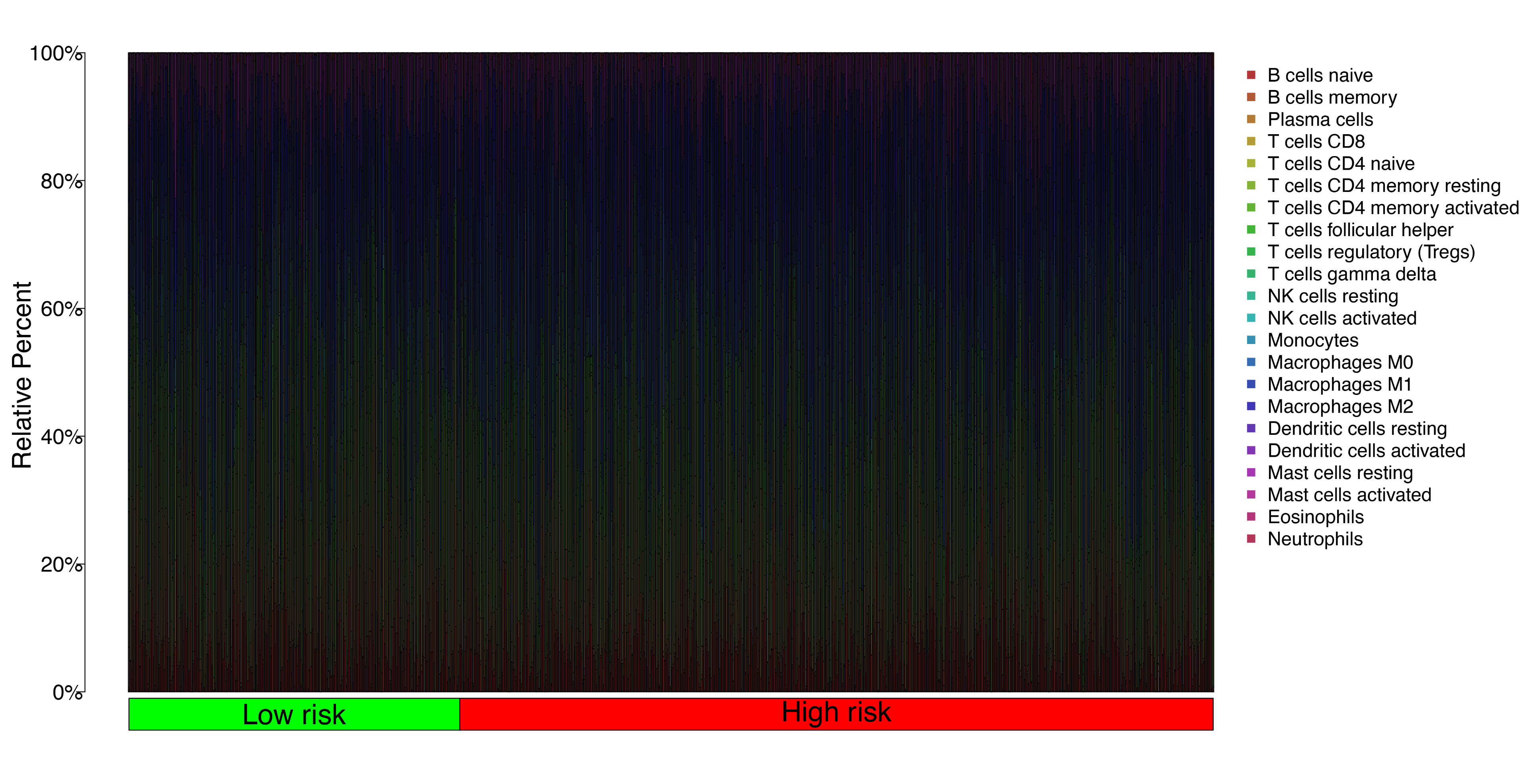

Supplement: Supplementary file 1 [file molecules-28-03283-s001.zip › Figure S5.tif]

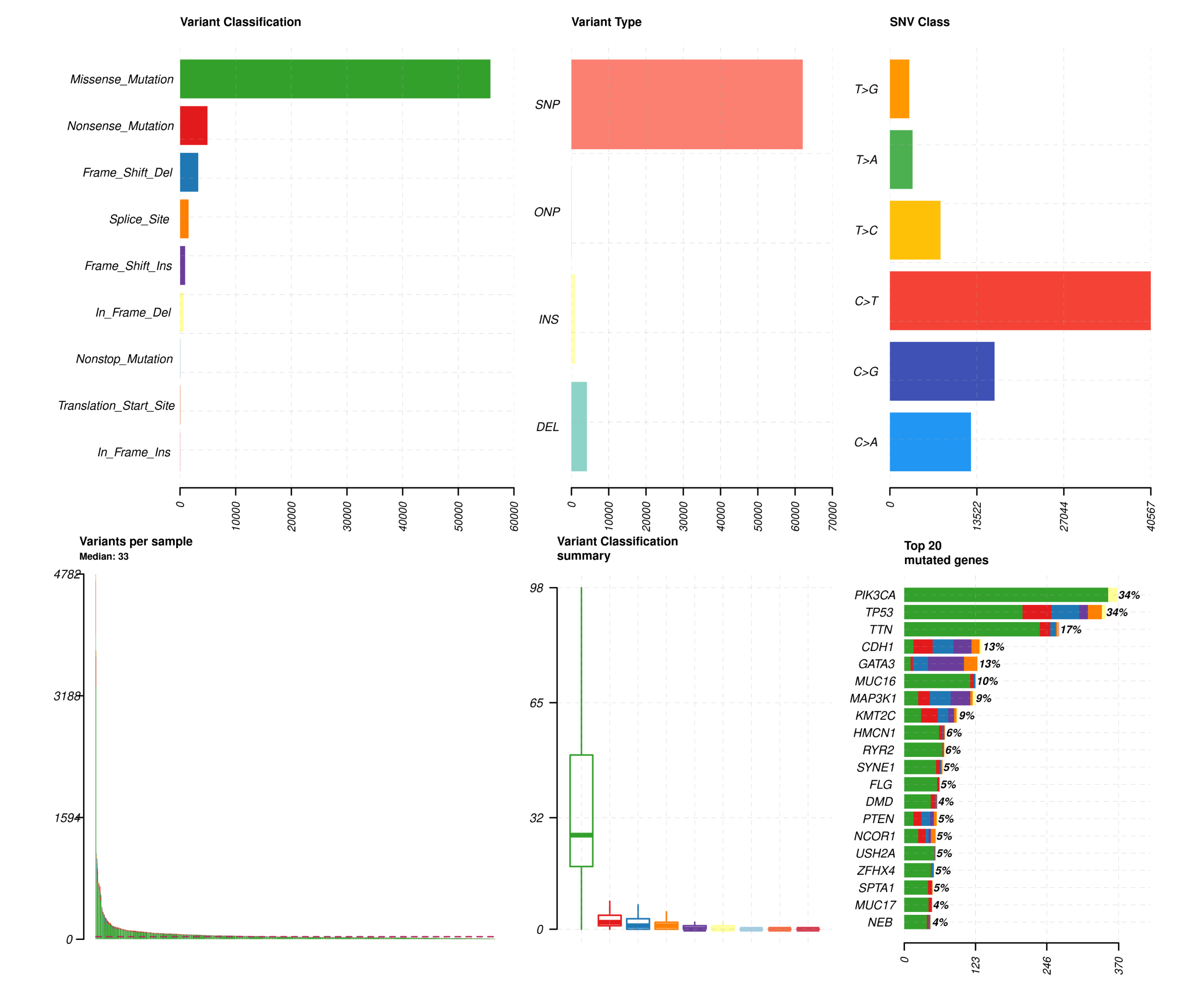

Supplement: Supplementary file 1 [file molecules-28-03283-s001.zip › Figure S6.tif]
